# Effect of Combined Visual Biofeedback and Metronome on Gait Reciprocity in Patients with Ischemic Stroke: A Pilot Study

**DOI:** 10.3390/bioengineering13080851

**Published:** 2026-07-23

**Authors:** Dmitry Skvortsov, Aliya Khudaigulova, Danila Lobunko, Sergey Kaurkin, Galina Ivanova

**Affiliations:** 1 Federal State Budgetary Institution “Federal Center of Brain Research and Neurotechnologies” of the Federal Medical Biological Agency, Moscow 117321, Russia; 2 FNKC FMBA, Research and Clinical Centre of Moscow, Moscow 107031, Russia

**Keywords:** stroke, hemiparesis, gait, biofeedback, rehabilitation

## Abstract

Background. Visual biofeedback (BF) and rhythmic metronome stimulation (RMS) are used for correction of stroke gait. To determine the feasibility of improving gait reciprocity using BF technology combined with RMS. Methods. Patients with subacute stroke in the Main group (n20 patients) underwent a course of BF training targeting the reciprocity parameter by RMS (9 sessions) plus standard rehabilitation. The Control group (n = 20) received standard rehabilitation only. Biomechanical gait analysis covering spatiotemporal, kinematic, and EMG parameters, as well as clinical scale assessments, was performed before and after the rehabilitation. Results. At baseline and at the end of rehabilitation, the Main group showed a functionally more severe condition on the DGI and TUG scales (*p* < 0.05). Both groups displayed a typical picture of hemiparetic gait with asymmetry of biomechanical, kinematic, and EMG parameters. By the end of rehabilitation, the Main group showed a positive trend in the reciprocity parameter that did not reach statistical significance. In the Control group, no changes in the parameter were observed. Conclusions. A short-term (9-session) course of BF training did not produce a statistically significant improvement in the reciprocity parameter and requires confirmation in a powered randomized trial.

## 1. Introduction

According to a 2024 systematic review, the observed decline in stroke mortality and severity is offset by the increasing prevalence of stroke driven by the growing burden of modifiable risk factors. This trend, in turn, leads to higher healthcare expenditures on primary and secondary prevention, acute care, and long-term rehabilitation [1].

According to a 2021 study, inpatient stroke rehabilitation accounts for approximately USD 70,000; nevertheless, such expenditure does not guarantee a favorable outcome [2]. Available evidence indicates that one year after rehabilitation most patients have not recovered their prior quality of life despite receiving specialized care in recovery facilities, and they remain dependent on caregiver assistance [3]. The implementation of various technologies helps reduce treatment costs and shorten recovery times. One such technology is the biofeedback (BF) method [4,5,6].

BF technologies in stroke rehabilitation have been widely used since the 1980s. BF training is employed to restore gait automaticity—specifically through changes increasing joint range of motion [7], enhancing electromyographic (EMG) muscle activity [8], and correcting spatiotemporal asymmetry [9]. This approach has proven to be an effective tool for rehabilitation specialists, with minimal contraindications, making it suitable for use at virtually any stage of rehabilitation [9,10].

BF is widely used for gait rehabilitation in patients following stroke [8,10,11]. Various BF-integrated rehabilitation devices [5], including portable devices [12], are being developed. The applications of BF in restoring gait pattern are diverse and include improving walking speed, increasing joint range of motion and muscle strength, and correcting spatiotemporal gait asymmetry to improve reciprocal limb movement [4,13,14]. Multimodal BF has been shown to be effective in the rehabilitation of gait disorders after stroke [15].

Correction of post-stroke gait asymmetry is of key importance: in 50% of patients with hemiparesis, impairments related to reduced reciprocity persist [15]. In particular, impaired reciprocity may predominantly involve temporal or spatial parameters, or both [16,17]. Each case requires targeted correction—specifically, differences in step length, Stance Phase asymmetry, and earlier onset of the second double-support phase on the paretic limb compared with the intact limb all contribute to movement asymmetry. This increases the energy cost of walking, reduces stability of the paretic leg, places additional load on the non-paretic side and raises the risk of falls [18]. Restoring coordinated alternating limb activity (i.e., reciprocity) is therefore one of the key objectives of rehabilitation [19]. Impaired gait reciprocity can be corrected even in severe cases [20]. It is important to account for the stroke period, the location of the brain lesion [21], the state of cognitive functions, preserved hearing, and the ability to reproduce an appropriate gait rhythm—which deteriorates in stroke patients [22]. Patients should also be aware of the objectively measurable asymmetry of the locomotor pattern, since individuals with chronic gait disorders resulting from prolonged compensatory adaptations have a substantially diminished ability to recognize impaired reciprocity as a kinematic error [23].

Despite the promising effectiveness and widespread use of BF technologies for improving post-stroke gait reciprocity, questions remain regarding their use in the context of short hospital stays, the need for specialized equipment, and the variability in skill transfer to daily activities. Moreover, most studies focus on long-term recovery programs [24,25]. Therefore, the potential of short-term intensive interventions remains insufficiently studied.

Objective: To evaluate the effectiveness of BF combined with metronome cueing for improving gait reciprocity in patients with subacute ischemic stroke during a 14-day intervention.

## 2. Materials and Methods

### 2.1. Study Design

Experimental, randomized, interventional, controlled longitudinal, pilot. The study included 2 groups of patients with hemiparesis following first-ever ischemic stroke. The experimental group comprised 20 patients who received, in addition to conventional therapy, a 9-session BF training course to improve gait reciprocity. The Control group consisted of 20 patients who underwent conventional therapy only. Eligible patients were randomly assigned to the two groups.

Baseline characteristics of the intervention and Control groups are presented in Table 1. The groups did not differ on any of the assessed variables.

Registration: ClinicalTrials.gov identifier: NCT06299943. Registered 19 July 2021.

### 2.2. Patients

Inclusion Criteria: Hemiparesis following first-ever ischemic stroke; age up to 75 years; functional readiness for upright positioning; adequate orthostatic tolerance; independent ambulation (use of assistive devices was permitted); sufficient level of consciousness to follow instructions during assessment and training; absence of cognitive impairments preventing comprehension of the study tasks; absence of sensorimotor aphasia; absence of severe comorbid medical conditions, ischemic ECG changes, or heart failure (Killip class II or higher); absence of CNS or peripheral nervous system diseases other than stroke associated with neurological deficits (sequelae of trauma, tumors, polyneuropathies, etc.); absence of orthopedic disorders (joint deformities and contractures, severe pain, limb amputations, etc.).

Exclusion Criteria: Persistent inadequate cardiovascular response during training; fear of treadmill walking; patient refusal to participate in therapeutic procedures; neurological and/or somatic clinical deterioration during rehabilitation; previous botulinum toxin treatment; recurrent stroke; mild hemiparesis not significantly affecting walking function; biomechanical gait parameters close to normal; hearing impairment.

The standard inpatient rehabilitation course includes nine days of active rehabilitation. The daily program lasted at least three hours. After assessing motor status using clinical scales and identifying impairments, a multidisciplinary team determined physical therapy methods on the day of admission: exercise machines, coordination and balance equipment, virtual reality systems, vibration therapy, and reflexology. Unlike the Intervention group, the Control group performed treadmill training without BF. All sessions were supervised by a physical therapist, who provided assistance to patients as needed. Upon completion of the course, immediately before discharge, the patients were reassessed to determine progress and any changes.

Patients in both groups underwent rehabilitation care in a rehabilitation unit for two weeks. Assessments were performed twice: on the day of admission and on the day of discharge.

The study was conducted in the biomechanics laboratory of the Research Center for Medical Rehabilitation, Federal Center for Brain and Neurotechnologies, Federal Medical and Biological Agency of Russia, from August 2025 to February 2026.

### 2.3. Clinical Methods

Clinical Scales. Gait-evaluating clinical scales: Timed Up and Go Test (TUG) [26], Hauser Ambulation Index (HAI) [27], 10-Meter Walk Test (10 MWT) [28], Dynamic Gait Index (DGI) [29]. Lower-limb muscle strength was assessed using the Medical Research Council Weakness Scale (MRC) [30] and categorized according to the International Classification of Functioning, Disability, and Health (ICF) [31].

### 2.4. Biomechanical Gait Analysis

Two biomechanical assessments were performed to objectively evaluate gait function: one before the start of rehabilitation and one after completion of the BF-therapy course and therapeutic interventions. Assessments were performed using the Steadys system (Neurosoft, Ivanovo, Russia). The Neurosens kit, consisting of 7 inertial sensors, was used. The inertial sensors transmitted the recorded data to a computer via Wi-Fi. Sensors were secured with elastic straps over the following areas: sacrum, over the lateral aspect of the upper third of the thigh, lower third of the shank (at the level of the lateral malleoli), and the dorsal surface of both feet.

Each sensor had two EMG recording channels and collected data at a frequency of 2000 Hz. The EMG data were high-pass filtered at 30 Hz, rectified, and low-pass filtered at 6 Hz to create linear envelopes. The notch filter (50 Hz) was not used because the sensor operated on independent battery power. The EMG signal was recorded from the major lower-limb muscle groups using disposable surface Mederen electrodes (Jaffa, Israel) fixed to the skin. Maximum electrical activity in microvolts (μV) was measured for: tibialis anterior (TA), triceps surae (TS), quadriceps femoris (QF), and hamstring muscles (HM). The selection of the flexor–extensor muscles of the lower leg and hip was limited only by the fact that, in accordance with the inclusion criteria, patients were already able to move independently and did not even use assistive devices.

After all sensors and electrodes were secured, the patient was asked to assume a neutral stance for calibration: standing upright, feet shoulder-width apart, arms alongside the trunk, hips and knees extended, gaze directed forward. Biomechanical parameters were recorded during walking at the patient’s preferred pace over a 10 m course, with reversal at the end of each pass. The software (SteadysPlus-2.1.0.0) automatically excluded steps with unstable parameters (acceleration, deceleration, stops) and processed all remaining gait cycles. Recording continued until 40 gait cycles were obtained for each leg. A trained neural network identified the gait cycle (GC) for the right and left legs and computed the remaining gait parameters [32].

The following biomechanical parameters were selected for analysis: temporal: GC duration (s), rhythm coefficient (RC); individual GC time periods expressed as a percentage of the GC: Stance Phase (SP), Single Support Phase (SSP), Double Stance Phase (DSP), and the reciprocity parameter—the Beginning of the Terminal Double Limb Stance Phase (BTDLS); spatial: foot clearance (cm), circumduction amplitude (cm), and walking speed (V, km/h). Kinematic parameters were recorded at 200 Hz frequency for the hip, knee, and ankle joints in the sagittal plane (flexion–extension) by constructing a goniogram across the GC with subsequent analysis of maximal motion amplitudes in degrees.

The linear envelopes recorded during each trial were averaged over the duration of the gait cycles. The EMG linear envelopes of each monitored muscle and goniograms of joints were downloaded to a spreadsheet. Each EMG linear envelope and each goniogram of joints contained 200 points per gait cycle. The final report included averaged EMG and joint-angle curves representing the mean gait cycle across 40 gait cycles, normalized to 200 points for each graph. This normalized the data and allowed them to be processed in a common time base.

The authors used a technique for muscle electrode placement, differing from the SENIAM standard. The study sought to obtain EMG from large muscle groups, rather than individual muscles or parts of them. Therefore, electrodes were placed transversely on the thigh and lower leg [33].

### 2.5. BF Training Protocol

The Steadys system allows BF training for any target biomechanical gait parameter. In this study, the target parameter was reciprocity (BTDLS). At the start of each training session, BTDLS was assessed using two inertial sensors secured with elastic straps at the level of the lateral malleoli. The patient was positioned on the treadmill, and a comfortable speed was set. The session was then performed at the patient’s self-selected comfortable walking speed. A two-minute biomechanical assessment was performed, after which a report with spatiotemporal gait parameters was generated, highlighting parameters outside the normal range.

**Figure 1:**
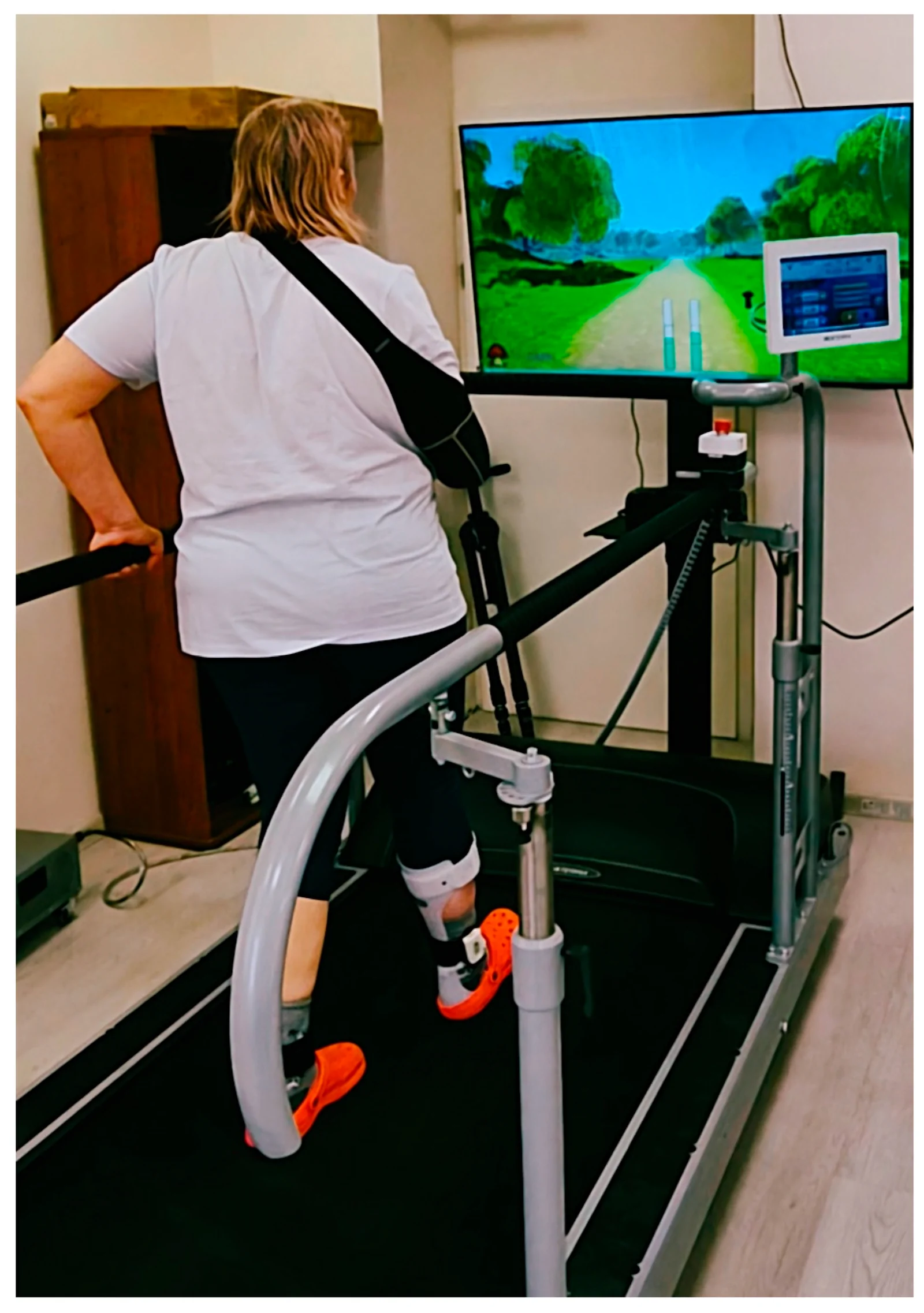
Biofeedback training.

The target parameter—BTDLS—was then set. The patient selected a virtual environment. The BF training procedure (Figure 1) then began. A virtual environment with two columns, each corresponding to the selected parameter for the left and right leg, was displayed on a screen in front of the patient. A visible acceptable range was defined; excursions beyond the corridor were recorded by the software as errors. This impeded the patient’s progress through the virtual environment and served as a cue for correct task execution. The training algorithm was designed so that, upon successful task completion, the acceptable range of values automatically shifted toward greater parameter symmetry. Initially, the range boundaries were set automatically but could be adjusted manually as needed.

The metronome tempo was then calibrated to the individual step frequency determined during the initial assessment at the start of the session. In this way, visual BF was combined with the use of a metronome set to the patient’s step frequency. The metronome was activated one minute after the patient started walking in the virtual environment. The metronome’s start time coincided with the beginning of the support period of every gait cycle. This facilitated the patient’s adaptation to metronome cueing. Prior to this, the patient underwent preliminary training during which the participant learned to begin the gait cycle exactly on the beat of the metronome. If the patient lost rhythm, the operator provided necessary instructions. However, such incidents were rare and were corrected within 2–3 gait cycles.

The Intervention group completed 9 training sessions. The duration of each session depended substantially on the patient’s functional status at the time. The criterion for ending a session was the appearance of fatigue signs: autonomic reactions, dyspnea, and frequent errors that could not be corrected. The biomechanical marker of fatigue was increasing reciprocity asymmetry. For more precise monitoring, the operator could view real-time values of the principal biomechanical parameters. The mean training duration was 26.8 ± 2.4 min (range 19–30 min). All training sessions were performed in one session without breaks.

The treadmill used was a ReaTerra (Moscow, Russia). Walking speed was adjusted according to the patient’s subjective responses and training safety criteria (always using handrails). The mean walking speed was 1 ± 0.2 km/h (range 0.7–1.5 km/h).

### 2.6. Statistical Analysis

Data were analyzed using Statistica 12.0. The Shapiro–Wilk test for normality of quantitative variables showed that distributions deviated from normal (*p* < 0.05). Data are presented as medians and first and third quartiles. Within-group comparisons were performed using the Wilcoxon test; between-group comparisons used the Mann–Whitney U-test. A *p* value < 0.05 was considered statistically significant.

## 3. Results

Results of the dynamics assessed by gait-evaluating scales are presented in Table 2.

After treatment, statistically significant improvement was observed on all scales in both groups. The Intervention group showed significantly higher severity scores on the DGI and TUG both before and after treatment compared with the Control group (*p* < 0.05).

Data on MRC changes and ICF categories related to gait function are presented in Table 3.

ICF codes b770, d4551, and d4500 significantly reflected greater clinical severity in the Main group compared with the Control group before the start of treatment. After treatment, this tendency persisted for codes b770 and d4500. Significant improvement was observed in all ICF scores, indicating reduced activity limitations in both groups after completion of the rehabilitation course (*p* < 0.05). Comparison of groups on the MRC revealed no differences.

Results of the spatiotemporal and gait-phase parameter analysis are presented in Table 4.

The initial assessment of both groups revealed the following findings: within-group comparison on the paretic side and contralateral limbs showed reduced foot clearance, increased circumduction amplitude, shorter SP, and earlier onset of SSP and BTDLS compared with the corresponding values on the contralateral side. Between-group comparison of baseline parameters of the paretic and contralateral sides of the Intervention group with the corresponding values for both limbs of the Control group revealed prolonged GC duration and increased circumduction in the Main group.

Following the BF training, the Intervention group demonstrated: within-group comparison of the paretic limb showed later onset of SSP and improved RC. On the contralateral side, shortening of SP was observed.

Analysis of the Control group showed shortening of DSP of both limbs and an increase in walking speed following conventional therapy.

After rehabilitation, within-group analysis of both groups again showed reduced foot clearance, increased circumduction amplitude, shorter SP, and earlier onset of SSP and BTDLS on the paretic side compared with the contralateral side. Post-rehabilitation between-group comparison of the paretic and contralateral sides of the Intervention group with the corresponding values for both limbs of the Control group continued to show prolonged GC duration and increased circumduction in the Intervention group.

Kinematic parameters for the hip, knee, and ankle joints are presented in Table 5.

The initial biomechanical assessment of the Intervention group demonstrated the following statistically significant differences in kinematic parameters: reduced hip and knee range of motion on the paretic side compared with the contralateral side. Analysis of the Control group baseline data showed reduced amplitude in all evaluated joints compared with the opposite side.

The follow-up assessment revealed: increased hip amplitude bilaterally and increased knee amplitude on the paretic limb in the Intervention group, and reduced hip amplitude on the contralateral side in the Control group at discharge.

Compared with the Control group, the Intervention group demonstrated greater hip range of motion and lower ankle range of motion.

**Figure 2:**
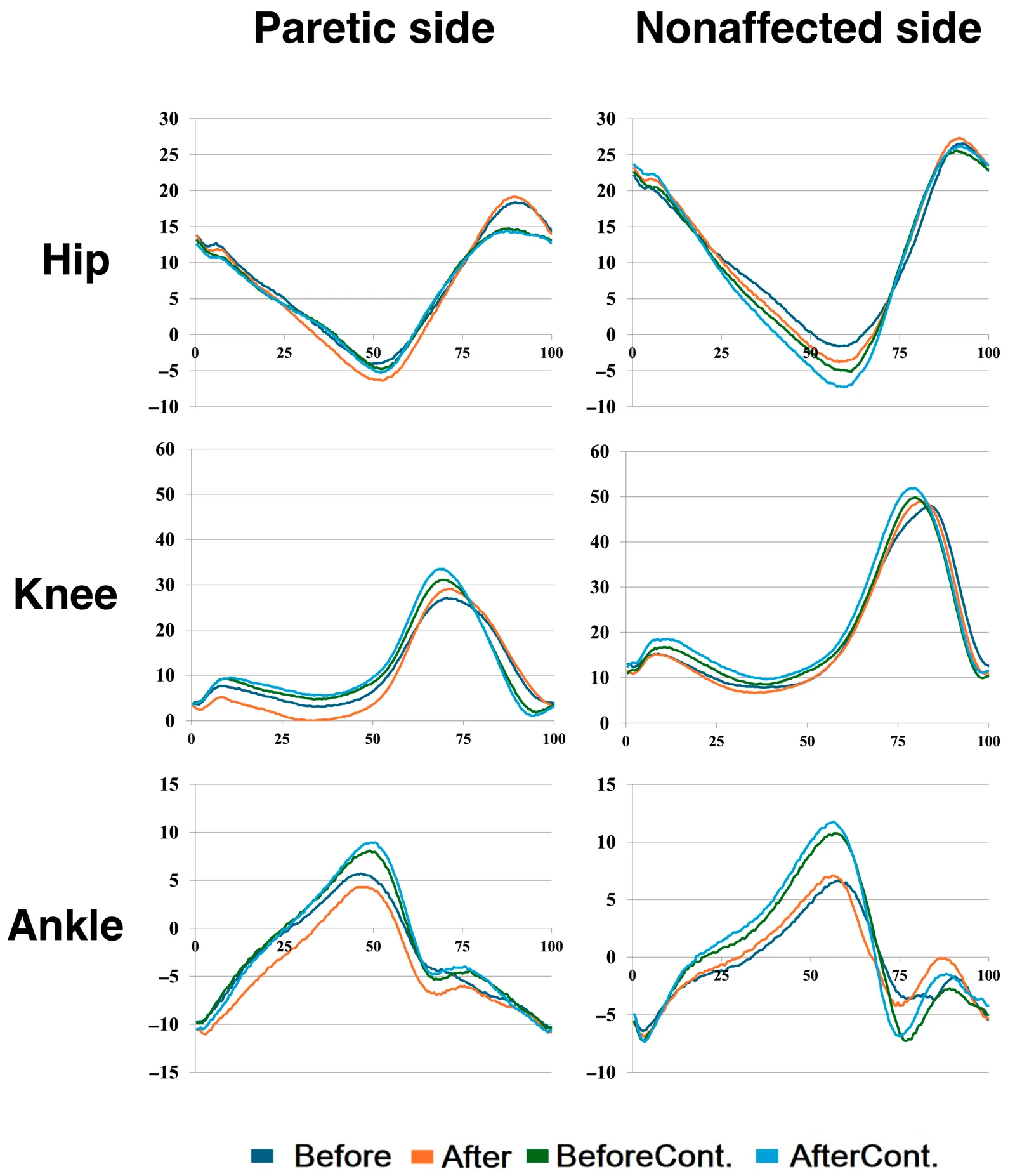
Goniograms of the hip, knee, and ankle joints for both groups. The vertical scale is in degrees; the horizontal scale is in % of the gait cycle.

The goniograms of joint motion (Figure 2) reveal additional phenomena not captured by analysis of maximal amplitudes.

Two clear phenomena are apparent. First, the goniograms demonstrated substantially greater severity of impairment in the Intervention group, as illustrated by the paretic-side goniograms: maximal amplitudes for all three joints in the Intervention group are lower than in the Control group. On the healthy side, this is also well demonstrated for the ankle joint. Second, across the rehabilitation period, joint function in each group changed very little.

EMG activity of the studied muscles is presented in Table 6.

Bioelectric muscle activity in the Intervention group showed reduced TA, TS, and HM on the paretic side before treatment compared with the contralateral side. Analysis of the Control group revealed reduced activity in all evaluated muscle groups compared with the contralateral side.

Assessment of changes in both groups revealed: increased TS activity on the paretic limb in the Intervention group; increased TA activity and reduced QF activity on the intact side in the Control group.

Analysis of post-treatment data in the Intervention group showed lower TS, HM, and QF compared with the contralateral side. Similarly, the Control group continued to demonstrate reduced EMG activity in all evaluated muscles on the paretic side compared with the contralateral side.

**Figure 3:**
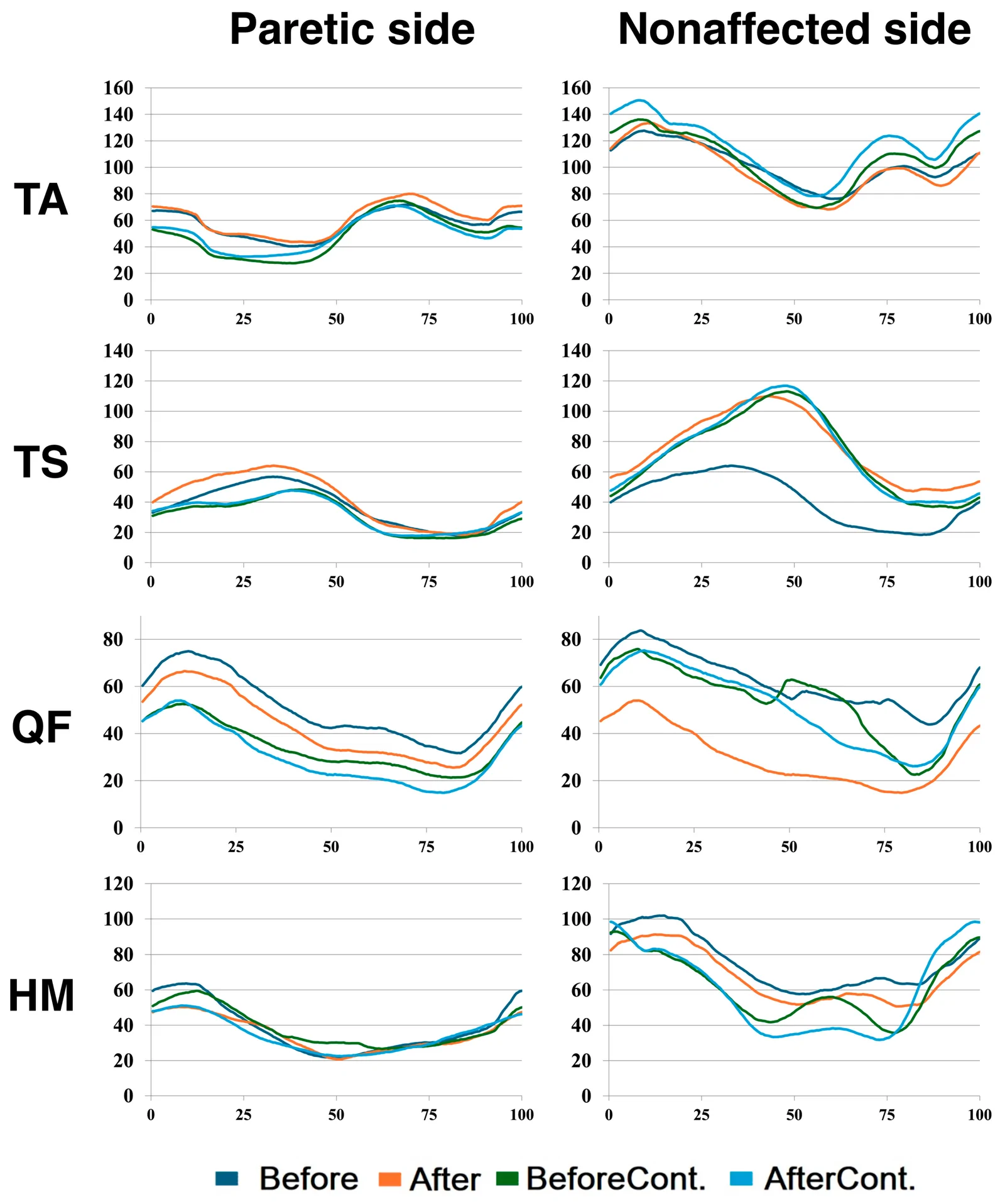
EMG envelope during gait cycle: tibialis anterior (TA), triceps surae (TS), quadriceps femoris (QF), and hamstring (HM). The vertical scale is in μV; horizontal—% of gait cycle.

Bioelectric muscle activity profiles of the Main group as a function of the GC are illustrated in Figure 3.

To a lesser extent, both phenomena noted for joint kinematics were also present for the paretic-side muscles.

## 4. Discussion

Analysis of baseline clinical scale data evaluating gait function showed that the Intervention group demonstrated poorer gait performance on the DGI and TUG at treatment onset. These differences persisted through the end of the rehabilitation course. Biomechanical analysis confirmed differences at the level of joint kinematics.

The pattern of changes in biomechanical, kinematic, and EMG parameters is consistent with the typical hemiparetic gait pattern [34,35]. The asymmetry of gait parameters in hemiparesis shows statistically significant differences between the paretic and contralateral sides in both groups. Specifically, shorter SP, earlier onset of SSP and BTDLS are characteristic of the paretic limb. Longer SSP and later onset of SSP and BTDLS characterize the intact side. All principal changes persisted after the two-week rehabilitation course in both groups.

The reciprocity parameter—the primary training target for the Intervention group—approached normal values after the BF training course, but the difference did not reach statistical significance (*p* = 0.15). The BTDLS in the Control group remained unchanged. Thus, the BTDLS parameter trained using BF did not show a statistically significant change. Comparable training approaches have proven effective in similar studies. A 2022 study compared outcomes of two groups after four weeks of rehabilitation [5]: the Main group received, in addition to standard rehabilitation, 10 min sessions of visual BF treadmill therapy targeting step length, walking speed, and SSP of the paretic limb. In the Control group, the functional therapy session was replaced by exercises guided by a physiotherapist. The outcome measures improved significantly in the Intervention group compared with the Control. A similar study by Jo YJ et al. [36] focused patient attention on SSP of the paretic limb—visualized on a screen as a real-time game—to correct step-length asymmetry and RC. After four weeks of treatment, the Main group achieved improvement in RC and paretic-limb step length compared with the Control. However, both studies used a four-week training program. Our study lasted only two weeks, comprising nine sessions, which we hypothesize was insufficient to produce the necessary effect in patients with subacute stroke. This hypothesis should be investigated in future studies. Our previous study [37] of a single, short training session on the BTDLS demonstrated that BF training may be effective, leading to a measurable change in the parameter. Two-week visual BF training can be effective and contributes to shortening of SSP, increasing swing phase, and lengthening GC on the healthy limb [38]; however, that result was obtained in patients in the late stroke period, when the main processes of motor function recovery had already occurred.

Combined BF technologies—paired with vibro-stimulation, electrical stimulation, or rhythmic auditory stimulation—proved more effective than isolated approaches [39,40,41]. Combining BF training with rhythmic auditory cues to promote gait symmetry over an 8-week course [41] was associated with a positive result in the form of improved spatiotemporal gait parameters and balance. Importantly, this was achieved after a 2-month course in patients with chronic stroke. A shorter 3-week course of visual BF combined with rhythmic auditory stimulation versus isolated auditory stimulation [42] also demonstrated the effectiveness of this approach, but again for patients in the chronic stage. Moreover, combining rhythmic auditory stimulation with vibrotactile feedback proved significantly more effective than isolated auditory stimulation [43].

In this study, a standard smartphone metronome was used. Two patients noted that the metronome sometimes impeded navigation through the virtual environment, distracting them and disrupting their already habitual asymmetric gait pattern with a steady beat. Conversely, some participants preferred to synchronize with the metronome, using the BF mainly as a correctness marker and only occasionally glancing at the screen. In addition, all patients experienced additional cognitive load—they reported greater fatigue during our training compared with other physical activities (stationary cycling, balance training device, physical therapist sessions).

It must be noted that shortening the inpatient stay of subacute stroke patients to two weeks in our institution adversely affects gait function recovery. Even with intensive multimodal intervention incorporating all methods included in the specialized care standard along with cutting-edge technologies, the short-term rehabilitation process failed to produce a stable change in the locomotor pattern according to biomechanical assessment (as opposed to improvement on clinical scales).

Despite the results suggesting the potential of combining BF therapy with rhythmic auditory stimulation, the present study has several limitations. First, the relatively small sample size and variability in patient clinical characteristics reduce the statistical power of the analysis, which likely accounts for the absence of statistically significant differences despite visually apparent improvement in reciprocity parameters. Second, the limited duration of the rehabilitation course did not allow a full assessment of the stability and clinical significance of the changes in gait biomechanical parameters, particularly BTDLS. Third, the absence of long-term follow-up does not permit conclusions about the persistence or attenuation of the effect achieved in the long term.

## 5. Conclusions

BF training combined with rhythmic auditory metronome stimulation in patients with subacute stroke did not demonstrate sufficient effectiveness when administered in a course of nine daily sessions over two weeks.

Future studies should be conducted with a substantially larger number of participants and training days.

## Figures and Tables

**Table 1:** Characteristics of the study groups.

Parameter	Main, n = 20		Control, n = 20	
Sex	Male	17	Male	15
	Female	3	Female	5
Age (years)	61 [57; 64]		56 [49; 63]	
Height (m)	1.77 [1.7; 1.82]		1.7 [1.65; 1.78]	
Weight (kg)	80 [74; 90]		75 [72; 87]	
Hemisphere	Right	12	Right	8
	Left	8	Left	12
Days since stroke	143 [110; 193]		152 [85; 203]	

**Table 2:** Assessment by scales evaluating gait function.

Scale	Main		Control	
	Before	After	Before	After
DGI	14 # [13; 15]	17 *# [15; 18]	17 [14; 18]	19 * [18; 20]
HAI	4 [4; 5]	4 * [4; 4]	4 [4; 4]	4 * [3; 4]
TUG (s)	36 # [25; 41]	26 *# [20; 35]	21 [17; 29]	18 * [14; 20]
10 MWT (m/s)	1 [1; 1]	1 * [1; 1]	1 [1; 1]	1 * [1; 1]

“Before”—parameter before the BF training course; “After”—parameter after the BF training course. * *p* < 0.05 vs. the same parameter before treatment; # *p* < 0.05 vs. the Control group.

**Table 3:** MRC and ICF domains.

Parameter	Main		Control	
	Before	After	Before	After
Muscle Group				
Hip flexors	3 [3; 4]	3 [3; 4]	4 [3; 4]	4 [3; 4]
Hip extensors	3 [3; 4]	3 [3; 4]	4 [3; 4]	4 [3; 4]
Knee flexors	3 [3; 4]	3 [3; 4]	3 [3; 4]	3 [3; 4]
Knee extensors	3 [3; 4]	3 [3; 4]	3 [3; 4]	3 [3; 4]
Ankle flexors	2 [1; 3]	2 [1; 3]	3 [2; 3]	3 [2; 3]
Ankle extensors	2 [1; 3]	2 [1; 3]	3 [2; 3]	3 [2; 3]
ICF Codes				
b770 Gait pattern functions	2 [2; 2] #	2 [1; 2] *#	2 [1; 2]	1 [1; 2] *
d4551 Climbing	3 [2; 3] #	2 [1; 2] *	2 [1; 2]	1 [0; 2] *
d4500 Walking short distances	2 [2; 2] #	1 [1; 2] *#	1 [1; 2]	1 [0; 1] *
d4501 Walking long distances	3 [2; 3]	2 [2; 3] *	3 [3; 3]	2 [2; 2] *

“Before”—parameter before the BF training course; “After”—parameter after the BF training course. * *p* < 0.05 vs. the same parameter before treatment; # *p* < 0.05 vs. the Control group.

**Table 4:** Spatiotemporal parameters and gait phases.

Parameter	Point	Main		Control	
		Paretic	Non-Affected	Paretic	Non-Affected
GC (s)	Before	1.9 # [1.6; 2.5]	1.9 # [1.6; 2.5]	1.5 [1.3; 1.7]	1.5 [1.4; 1.7]
	After	1.9 # [1.6; 2.3]	1.9 # [1.6; 2.5]	1.4 [1.4; 1.7]	1.5 [1.4; 1.7]
RC (units)	Before	0.69 [0.61; 0.74]		0.73 [0.62; 0.85]	
	After	0.75 * [0.67; 0.87]		0.75 [0.6; 0.9]	
V (km/h)	Before	1.26 [0.86; 1.99]		1.7 [1.2; 2.28]	
	After	1.41 [1; 1.95]		2.05 * [1.48; 2.63]	
Clearance (cm)	Before	9 $ [8; 11]	12 [11; 13]	10 $ [7; 11]	11 [10; 12]
	After	10 $ [8; 12]	12 [11; 13]	10 $ [8; 11]	12 [11; 13]
Circumduction (cm)	Before	7 #$ [5; 8]	4 # [3; 4]	3 $ [3; 5]	3 [2; 3]
	After	6 $# [5; 8]	3 # [3; 5]	4 $ [3; 5]	2 [2; 3]
SP (%)	Before	64.8 $ [61.7; 72.2]	76.1 [71.8; 82.9]	63.9 $ [61.6; 66.4]	74.9 [69.6; 76.8]
	After	66.1 $ [62.3; 74.1]	74.7 * [72.2; 79.9]	63 $ [61.7; 66]	72.8 [69; 77.6]
SSP (%)	Before	23.6 $ [19.2; 28.6]	34.9 [29.7; 38.1]	25.1 $ [22; 31.3]	36.2 [34.2; 37.9]
	After	25.7 *$ [20.9; 28.2]	34.1 [28; 37.6]	26.8 $ [23.7; 31.4]	37.2 [34.5; 38.1]
DSP (%)	Before	39.7 [34; 53.2]	39.8 [33.9; 53.6]	38 [35; 41.4]	38 [35; 40.9]
	After	40.2 [32.7; 52.4]	40.4 [32.8; 51.1]	35.4 * [33.1; 40.1]	35.3 * [33.2; 40.1]
BTDLS (%)	Before	42.5 $ [36.2; 47.2]	58.3 [53.7; 65.4]	43.7 $ [41.1; 45.6]	56.2 [54; 58.4]
	After	45.8 $ [40.7; 48.5]	54.8 [52.2; 59.2]	44.1 $ [42.1; 46.6]	56.6 [53.8; 58.7]

“Before”—before the BF course; “After”—after the BF course. * *p* < 0.05 vs. the same parameter before treatment; # *p* < 0.05 vs. the Control group; $ *p* < 0.05 vs. the contralateral side.

**Table 5:** Parameters of amplitude of joint motion.

Joint	Point	Main		Control	
		Paretic	Non-Affected	Paretic	Non-Affected
Hip	Before	22 [19; 32] $	32 [29; 37]	21 [17; 27] $	33 [30; 35]
	After	25 [21; 33] *#$	35 [31; 40] *	20 [17; 27] $	32 [31; 40] *
Knee	Before	34 [26; 43] $	52 [45; 57]	35 [28; 42] $	50 [45; 54]
	After	35 [32; 53] *$	53 [51; 56]	36 [31; 45] $	53 [46; 57]
Ankle	Before	21 [16; 26]	23 [19; 26]	23 [21; 28] $	26 [22; 28]
	After	21 [16; 25] #	23 [19; 28]	25 [23; 29]	27 [24; 29]

“Before”—before the BF course; “After”—after the BF course. * *p* < 0.05 vs. the same parameter before treatment; # *p* < 0.05 vs. the Control group; $ *p* < 0.05 vs. the contralateral side.

**Table 6:** Maximum EMG amplitudes of the studied muscles.

Muscle	Point	Main		Control	
		Paretic	Non-Affected	Paretic	Non-Affected
TA (μV)	Before	93 [50; 139] $	162 [105; 216]	84 [56; 103] $	161 [100; 188]
	After	89 [59; 134]	159 [101; 196]	89 [70; 107] $	167 [141; 209] *
TS (μV)	Before	44 [32; 66] $	121 [91; 145]	50 [39; 69] $	139 [95; 155]
	After	53 [35; 98] *$	140 [113; 175]	50 [40; 70] $	124 [84; 154]
QF (μV)	Before	66 [44; 105]	88 [53; 128]	62 [41; 73] $	87 [71; 129]
	After	56 [45; 93] $	101 [53; 143]	54 [36; 76] $	71 [56; 105] *
HM (μV)	Before	79 [42; 99] $	102 [69; 160]	56 [42; 73] $	104 [82; 142]
	After	50 [32; 86] $	97 [58; 144]	45 [35; 69] $	95 [84; 120]

“Before”—before the BF course; “After”—after the BF course. * *p* < 0.05 vs. the same parameter before treatment; $ *p* < 0.05 vs. the contralateral side.

## Data Availability

The original contributions presented in this study are included in the article. Further inquiries can be directed to the corresponding author.

## Abbreviations

The following abbreviations are used in this manuscript:

BF	Biofeedback
EMG	Electromyographic
TUG	Timed Up and Go test
HAI	Hauser Ambulation Index
10 MWT	10-Meter Walk Test
DGI	Dynamic Gait Index
MRC	Medical Research Council Weakness Scale
ICF	International Classification of Functioning, Disability, and Health
TA	Tibialis anterior muscle
TS	Triceps surae muscle
QF	Quadriceps femoris muscle
HM	Gamstring
GC	Gait Cycle
RC	Rhythm coefficient
SP	Stance Phase
SSP	Single Support Phase
DSP	Double Stance Phase
BTDLS	Beginning of the Terminal Double Limb Stance Phase

## References

[1] (2024). GBD 2021 Stroke Risk Factor Collaborators. Global, regional, and national burden of stroke and its risk factors, 1990–2021: A systematic analysis for the Global Burden of Disease Study 2021. Lancet Neurol..

[2] van Meijeren-Pont W., Tamminga S.J., Goossens P.H., Groeneveld I.F., Arwert H., Meesters J.J.L., Mishre R.R., Vlieland T.P.M.V., van den Hout W.B. (2021). on behalf of the Stroke Cohort Outcomes of REhabilitation (SCORE) study group. Societal burden of stroke rehabilitation: Costs and health outcomes after admission to stroke rehabilitation. J. Rehabil. Med..

[3] Schindel D., Schneider A., Grittner U., Jöbges M., Schenk L. (2021). Quality of life after stroke rehabilitation discharge: A 12-month longitudinal study. Disabil. Rehabil..

[4] Santucci V., Alam Z., Liu J., Spencer J., Faust A., Cobb A., Konantz J., Eicholtz S., Wolf S., Kesar T.M. (2023). Immediate improvements in post-stroke gait biomechanics are induced with both real-time limb position and propulsive force biofeedback. J. Neuroeng. Rehabil..

[5] Kaźmierczak K., Warenčzak-Pawlicka A., Miedzyblocki M., Lisiński P. (2022). Effect of treadmill training with visual biofeedback on selected gait parameters in subacute hemiparetic stroke patients. Int. J. Env. Res. Public Health.

[6] Pinheiro C., Figueiredo J., Cerqueira J., Santos C.P. (2022). Robotic biofeedback for post-stroke gait rehabilitation: A scoping review. Sensors.

[7] Herrin K.R., Pan Y.T., Kesar T.M., Sawicki G.S., Young A.J. (2025). Robotic ankle exoskeleton and limb angle biofeedback for assisting stroke gait: A feasibility study. IEEE Robot Autom. Lett..

[8] Wang R., Zhang S., Zhang J., Tong Q., Ye X., Wang K., Li J. (2024). Electromyographic biofeedback therapy for improving limb function after stroke: A systematic review and meta-analysis. PLoS ONE.

[9] Tate J.J., Milner C.E. (2010). Real-time kinematic, temporospatial, and kinetic biofeedback during gait retraining in patients: A systematic review. Phys. Ther..

[10] Spencer J., Wolf S.L., Kesar T.M. (2021). Biofeedback for post-stroke gait retraining: A review of current evidence and future research directions in the context of emerging technologies. Front. Neurol..

[11] Kim Y.S., Song J.Y., Park S.H., Lee M.M. (2023). Effect of functional electrical stimulation-based mirror therapy using gesture recognition biofeedback on upper extremity function in patients with chronic stroke: A randomized controlled trial. Medicine.

[12] Bowman T., Gervasoni E., Arienti C., Lazzarini S.G., Negrini S., Crea S., Cattaneo D., Carrozza M.C. (2021). Wearable devices for biofeedback rehabilitation: A systematic review and meta-analysis. Sensors.

[13] Aiello E., Gates D.H., Patritti B.L., Cairns K.D., Meister M., Clancy E.A., Bonato P. (2005). Visual EMG biofeedback to improve ankle function in hemiparetic gait. Conf. Proc. IEEE Eng. Med. Biol. Soc..

[14] Cha Y., Kim Y., Hwang S., Chung Y. (2014). Intensive gait training with rhythmic auditory stimulation in individuals with chronic hemiparetic stroke: A pilot randomized controlled study. NeuroRehabilitation.

[15] Patterson K.K., Gage W.H., Brooks D., Black S.E., McIlroy W.E. (2010). Evaluation of gait symmetry after stroke: A comparison of current methods and recommendations for standardization. Gait Posture.

[16] Goldie P.A., Matyas T.A., Evans O.M. (2001). Gait after stroke: Initial deficit and changes in temporal patterns for each gait phase. Arch. Phys. Med. Rehabil..

[17] Sheffler L.R., Chae J. (2015). Hemiparetic gait. Phys. Med. Rehabil. Clin. N. Am..

[18] Balasubramanian C.K., Neptune R.R., Kautz S.A. (2009). Variability in spatiotemporal step characteristics and its relationship to walking performance post-stroke. Gait Posture.

[19] Lee S., Lee K., Song C. (2018). Gait training with bilateral rhythmic auditory stimulation in stroke patients: A randomized controlled trial. Brain Sci..

[20] Wright R., Masood A., MacCormac E., Pratt D., Sackley C., Wing A. (2013). Metronome-cued stepping in place after hemiparetic stroke: Comparison of a one- and two-tone beat. ISRN Rehabil..

[21] Shabbir M., Arshad N., Naz A., Waris M., Javed M.A. (2020). Comparison between right and left hemisphere lesion of stroke patients for functional gait assessment. Ann. Pak. Inst. Med. Sci..

[22] Patterson K.K., Wong J.S., Knorr S., Grahn J.A. (2018). Rhythm perception and production abilities and their relationship to gait after stroke. Arch. Phys. Med. Rehabil..

[23] Wutzke C.J., Faldowski R.A., Lewek M.D. (2015). Individuals poststroke do not perceive their spatiotemporal gait asymmetries as abnormal. Phys. Ther..

[24] McCue P., Del Din S., Hunter H., Lord S., Price C.I.M., Shaw L., Rodgers H., Rochester L., Moore S.A. (2020). Auditory rhythmical cueing to improve gait and physical activity in community-dwelling stroke survivors (ACTIVATE): Study protocol for a pilot randomised controlled trial. Pilot Feasibility Stud..

[25] Song G.B., Ryu H.J. (2016). Effects of gait training with rhythmic auditory stimulation on gait ability in stroke patients. J. Phys. Ther. Sci..

[26] Chan P.P., Si Tou J.I., Tse M.M., Ng S.S. (2017). Reliability and validity of the Timed Up and Go Test with a motor task in people with chronic stroke. Arch. Phys. Med. Rehabil..

[27] Hauser S.L., Dawson D.M., Lehrich J.R., Beal M.F., Kevy S.V., Propper R.D., Mills J.A., Weiner H.L. (1983). Intensive immunosuppression in progressive multiple sclerosis. N. Engl. J. Med..

[28] Watson M.J. (2002). Refining the ten-metre walking test for use with neurologically impaired people. Physiotherapy.

[29] Jonsdottir J., Cattaneo D. (2007). Reliability and validity of the Dynamic Gait Index in persons with chronic stroke. Arch. Phys. Med. Rehabil..

[30] Paternostro-Sluga T., Grim-Stieger M., Posch M., Schuhfried O., Vacariu G., Mittermaier C., Bittner C., Fialka-Moser V. (2008). Reliability and validity of the Medical Research Council (MRC) scale and a modified scale for testing muscle strength in patients with radial palsy. J. Rehabil. Med..

[31] World Health Organization (2001). International Classification of Functioning, Disability and Health. https://www.who.int/standards/classifications/international-classification-of-functioning-disability-and-health.

[32] Skvortsov D., Chindilov D., Painev N., Rozov A. (2023). Heel-Strike and Toe-Off Detection Algorithm Based on Deep Neural Networks Using Shank-Worn Inertial Sensors for Clinical Purpose. J. Sens..

[33] Criswell E., Cram J.R. (2010). Cram’s Introduction to Surface Electromyography.

[34] Skvortsov D.V., Kaurkin S.N., Grebenkina N.V., Ivanova G.E. (2025). Typical changes in gait biomechanics in patients with subacute ischemic stroke. Diagnostics.

[35] Lee Y., Kim G.B., Shin S. (2025). Association between lower limb strength asymmetry and gait asymmetry: Implications for gait variability in stroke survivors. J. Clin. Med..

[36] Jo Y.J., Kim D.H., Kim S., Kim J.H., Choi J.H., Park J.B., Baek Y.S., Park Y.G., Kim D.Y. (2023). Effect of anterioposterior weight-shift training with visual biofeedback in patients with step length asymmetry after subacute stroke. J. Pers. Med..

[37] Skvortsov D.V., Khudaigulova A.R., Ivanova G.E. (2026). Impaired Reciprocal Gait in Patients with Ischemic Stroke: A Single-Session Recovery Trial. J. Clin. Pract..

[38] Drużbicki M., Guzik A., Przysada G., Kwolek A., Brzozowska-Magoń A. (2015). Efficacy of gait training using a treadmill with and without visual biofeedback in patients after stroke: A randomized study. J. Rehabil. Med..

[39] Chen T., Yang J., Yan J., Ding X. (2025). Effect of biofeedback electrical stimulation combined with early intensive rehabilitation training on stroke rehabilitation. Am. J. Transl. Res..

[40] Uhm Y.H., Yang D.J. (2017). The effects of whole body vibration combined biofeedback postural control training on balance ability and gait ability in stroke patients. J. Phys. Ther. Sci..

[41] Shin J., Chung Y. (2022). The effects of treadmill training with visual feedback and rhythmic auditory cue on gait and balance in chronic stroke patients: A randomized controlled trial. NeuroRehabilitation.

[42] Park J., Kim B., Kim T. (2018). Effects of visual feedback and rhythmic auditory stimulation on walking of stroke patients induced by treadmill walking training. Phys. Ther. Korea.

[43] Kim S.J., Kim S.M., Jang S.H. (2025). Comparison of rhythmic auditory stimulation gait training with and without vibrotactile feedback on balance and gait in persons with stroke: A randomized controlled trial. Bioengineering.

